# Adoptive transfer of CD3^+^ T cells and CD4^+^ CD44^high^ memory T cells induces autoimmune pancreatitis in MRL/MpJ mice

**DOI:** 10.1111/jcmm.13537

**Published:** 2018-01-31

**Authors:** Luise Ehlers, Sarah Rohde, Saleh Ibrahim, Robert Jaster

**Affiliations:** ^1^ Department of Medicine II Division of Gastroenterology Rostock University Medical Center Rostock Germany; ^2^ Institute of Experimental Dermatology University of Lübeck Lübeck Germany; ^3^ Clinical Science Department College of Medicine American University of Sharjah Sharjah United Arab Emirates

**Keywords:** adoptive transfer, autoimmune pancreatitis, cell culture, mouse model, T cells

## Abstract

The immunopathogenesis of autoimmune pancreatitis (AIP) is poorly understood. Here, we have used MRL/MpJ mice, a model of spontaneous AIP, to address the role of cellular autoimmune processes in the initiation and progression of the disease. Therefore, different T cell subpopulations were adoptively transferred from sick to still healthy (but susceptible) MRL/MpJ mice. Unpurified splenocytes and CD3^+^ T cells both efficiently induced AIP, while CD4^+^ and CD8^+^ T cells alone, as well as splenocytes from healthy mice, were insufficient to trigger the disease. Strikingly, CD4^+^ CD44^high^ memory T cells, although transferred at lower numbers than other T cells, also induced AIP in recipient mice. Employing a modified experimental design, we also evaluated the effects of regulatory T cells (*T*_regs_) on the progression of AIP in already diseased mice. Under the given experimental conditions, there was no significant suppressive effect of adoptively transferred *T*_regs_ on pancreatic histopathology. The results of our studies suggest a key role of T cell‐mediated processes in murine AIP. The effects of CD4^+^ CD44^high^ memory T cells are in accordance with genetic studies of our group, which had previously implicated this cell type into the pathogenesis of AIP. In follow‐up studies, we will focus on the interplay of cellular and humoral autoimmunity in the context of AIP.

## 1 INTRODUCTION

Autoimmune pancreatitis (AIP) is a rare form of chronic pancreatitis (CP). It is a differential diagnosis of pancreatic carcinoma, because clinical symptoms of both entities are very similar.^1^ However, the specific therapeutic requirements differ: while surgery is the only potentially curative therapy for pancreatic carcinoma, patients suffering from AIP respond well to steroids,^2^ a treatment that is ineffective in the more common forms of CP.

Both, humoral and cellular immunity have been implicated into the pathogenesis of AIP.^3^ Nevertheless, the cellular and molecular basis of this disease remains largely unknown. The humoral immune response has been in focus recently, but still it is incompletely understood. Several autoantibodies, for example anti‐carbonic anhydrase‐II, anti‐lactoferrin and antitrypsinogen, have been found in the serum of AIP patients.^4^ However, whether autoantibodies have an active role in the pathogenesis of AIP or are an epiphenomenon remains unclear. Furthermore, for a subgroup of AIP cases, elevated levels of immunoglobulin (Ig)‐G4 in serum and dense infiltrates of IgG4‐positive plasma cells in the tissue are pathognomonic. This entity, termed AIP type 1, has recently been defined as the pancreatic manifestation of IgG4‐related disease (IgG4‐RD).^5^, ^6^, ^7^ In contrast, AIP type 2 is characterized by granulocyte epithelial lesions (GELs) and the absence of IgG4‐positive cells.^2^

Less is known about cellular immune processes in AIP, although T cells are the prevalent type of infiltrating immune cells in affected tissue.^8^, ^9^ Both, CD4^+^ and CD8^+^ T lymphocytes are present in pancreatic parenchyma of AIP patients,^3^ but CD4^+^ T cells predominate in tissue infiltrates. Increased production of interferon (IFN)‐γ by CD4^+^ T_helper_‐cells type 1 (Th1) has been proposed to promote AIP,^8^ and treatment with IFN‐γ strongly aggravated AIP in mice.^10^ On the other hand, cytokines promoting the development of Th2 have also been described in the context of AIP.^11^

The MRL/MpJ mouse model has proven useful to study the pathogenesis of AIP. These mice spontaneously develop an AIP at the age of about 6 months.^12^, ^13^ The murine AIP histopathologically resembles the human AIP type 1. The incidence and the severity of the disease are higher for female than for male mice and can be triggered using polyinosinic:polycytidylic acid (poly I:C).^14^

Using this mouse model in previous studies, we gained genetic evidence for an involvement of CD4^+^CD44^high^ memory T cells in the pathogenesis of AIP.^15^ Likewise, our studies implicated regulatory T cells (*T*_regs_) in the mediation of the therapeutic effects of rapamycin through a suppression of the effector T cell response.^9^

One characteristic of autoimmune diseases such as AIP is the possibility to adoptively transfer the disease from sick to healthy individuals in in vivo experiments. In 1992, Kanno et al^12^ showed that unpurified splenocytes may transfer AIP from sick MRL/MpJ mice to healthy (but susceptible) animals. In contrast, the transfer of serum failed to induce AIP in the recipient mice. Together, these data point to a key role of cellular autoimmunity and a dispensability of autoantibodies in the development of experimental AIP.

However, splenocytes consist of a variety of different lymphocytes and the specific populations necessary for the transfer are still unknown. Thus, the aim of this work was to gain mechanistic insights into processes of cellular autoimmunity in murine AIP. We have used the MRL/MpJ mouse model to perform adoptive transfer experiments. Different T cell subpopulations were transferred to identify those that are able to induce AIP or to inhibit progress of the disease.

Our data indicate that both adoptively transferred CD3^+^ T cells and CD4^+^CD44^high^ memory T cells are sufficient to induce AIP in susceptible MRL/MpJ recipient mice, suggesting key roles of these specific T cell subpopulations in the immunopathogenesis of murine AIP. Interestingly, the transfer of immunosuppressive *T*_regs_ failed to inhibit progress of AIP in spontaneously diseased MRL/MpJ mice.

## 2 MATERIALS AND METHODS

### 2.1 Mouse strain

MRL/MpJ mice were purchased from Charles River Laboratories (Sulzfeld, Germany). These mice spontaneously develop an AIP at an age of about 24 weeks.^12^ Animals were kept under specific pathogen‐free conditions at a 12 hours light/dark cycle with food and water ad libitum. Additionally, soaked food was provided over the 6 weeks period of investigation. All procedures were performed with adherence to the EU Directive 2010/63/EU for animal experiments and approved by the local governmental administrations (Landesamt für Landwirtschaft, Lebensmittelsicherheit und Fischerei Mecklenburg‐Vorpommern).

### 2.2 Isolation of splenocytes and cell culture conditions

Splenocytes were obtained from female MRL/MpJ mice. Young and adult mice (8.5 ± 0.2 and 41 ± 0.6 weeks old, respectively) served as spleen donors. The animals were sacrificed by an overdose of ketamine/xylazine, serum was collected using clot activator containing tubes (Sarstedt, Nümbrecht, Germany), and the pancreas, liver and kidneys were harvested and cryo‐ or paraffin‐embedded for further analyses. The spleen was kept on ice in splenocyte culture medium RPMI‐1640 (Biochrom, Berlin, Germany) supplemented with 10% foetal calf serum (FCS; Biochrom), 1% penicillin/streptomycin (Biochrom) and 50 μmol/L β‐mercaptoethanol (Merck, Darmstadt, Germany). Subsequently, the organ was forced through a 70 μm cell strainer (Greiner bio‐one, Kremsmünster, Austria) and incubated in 10 mL splenocyte culture medium containing 50 μg/mL DNase I (Roche Applied Science, Mannheim, Germany) for 10 minutes. The cell suspension was centrifuged (300× *g*, 10 minutes, 4°C), resuspended in 1 mL phosphate‐buffered saline (PBS) and incubated for 3 minutes on ice with 4 mL of ice‐cold 0.25 M NH_4_Cl solution to lyse erythrocytes. Lysis was stopped by adding 10 mL of culture medium and centrifugation (300× *g*, 10 minutes, 4°C). The cells were sowed at a density of 1 × 10^6^ cells/mL in cell culture flasks. Based on the protocol of Kanno et al,^12^ 25 μg/mL phytohaemagglutinin (PHA; Merck) was added to each flask. Cells were cultured for 3 days at 37°C in a 5% CO_2_ humidified atmosphere.

### 2.3 Adoptive transfer of unpurified splenocytes, CD3^+^ T cells, CD4^+^ T_helper_‐cells, CD8^+^ cytotoxic T cells and CD4^+^CD44^high^ memory T cells

On day 3 of splenocyte culture, cells were harvested and washed with PBS, pH 7.4. Unpurified splenocytes were either used directly for adoptive transfer or subjected to isolation of lymphocyte subpopulations. The following kits were used to isolate untouched cells by negative selection: CD3^+^ T cells: Pan T Cell Isolation Kit II, mouse (130‐095‐130; Miltenyi Biotec, Bergisch Gladbach, Germany); CD4^+^ T_helper_‐cells: CD4^+^ T Cell Isolation Kit, mouse (130‐104‐454; Miltenyi Biotec); CD8^+^ cytotoxic T cells: CD8^+^a T Cell Isolation Kit, mouse (130‐104‐075; Miltenyi Biotec); CD4^+^CD44^high^ memory T cells: Mouse Memory T Cell CD4^+^/CD62L^−^/CD44^high^ Column Kit (MCD44; R&D Systems, Minneapolis, MN, USA). All kits were carried out following the manufacturer's instructions.

As recipients, 8.1 ± 0.2 to 9.5 ± 0.5‐week‐old MRL/MpJ mice of the indicated gender were employed. One week prior to cell transfer, recipient mice were treated once with 200 mg/kg bodyweight cyclophosphamide to facilitate engrafting (as described by Kanno et al^12^). For adoptive transfer, cells were diluted in PBS, and 5 × 10^6^ cells (unpurified splenocytes, CD3^+^, CD4^+^ and CD8^+^ T cells) or 2 × 10^6^ cells (CD4^+^CD44^high^ T cells and another group of unpurified splenocytes) in a volume of 200 μL were injected into the tail vain of recipient mice. Mice without cell transfer served as controls. Table 1 shows the different mouse cohorts including information about the age of animals, type and number of transferred cells and the gender of all mice.

**Table 1:** Characteristics of the mouse cohorts

Cohort	Donors			Transferred cells	Number of transferred cells	Recipients		
	Sex	Age (weeks ± SEM)	AIP‐score ± SEM			Sex	Age (weeks ± SEM)	n
Control	‐	‐	‐	None	‐	Female	9.5 ± 0.5	10
1	Female	38 ± 0.6	3.0 ± 0.1	Unpurified splenocytes	5 × 10^6^	Male	8.7 ± 0.1	10
2	Female	40 ± 1.3	2.9 ± 0.2	Unpurified splenocytes	5 × 10^6^	Female	9.3 ± 0.2	10
3	Female	44 ± 1.9	3.0 ± 0.2	CD3^+^ T cells	5 × 10^6^	Female	9.1 ± 0.3	10
4	Female	43 ± 1.8	2.9 ± 0.1	CD4^+^ T cells	5 × 10^6^	Female	8.6 ± 0.3	10
5	Female	43 ± 1.9	2.9 ± 0.1	CD8^+^ T cells	5 × 10^6^	Female	8.1 ± 0.2	9
6	Female	37 ± 0.4	2.9 ± 0.1	Unpurified splenocytes	2 × 10^6^	Female	9.0 ± 0.1	9
7	Female	39 ± 1.2	3.0 ± 0.2	CD4^+^CD44^high^ T cells	2 × 10^6^	Female	8.6 ± 0.2	10
8	Female	8.5 ± 0.2	0.2 ± 0.2	Unpurified splenocytes	5 × 10^6^	Female	8.5 ± 0.3	6

**Figure 1:**
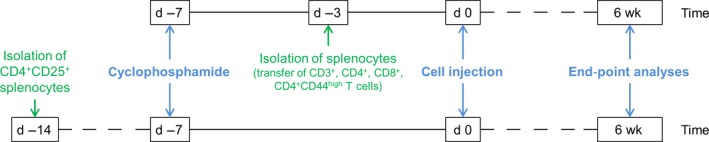
Workflow for adoptive transfer of different splenocyte subpopulations. Unpurified splenocytes were isolated from spleens of donor MRL/MpJ mice 14 days (*T*_regs_) or 3 days (CD3^+^, CD4^+^, CD8^+^, CD4^+^ CD44^high^ T cells) prior to cell injection. The subpopulations were isolated either on the same day (*T*_regs_) or right before the injection (CD3^+^, CD4^+^, CD8^+^, CD4^+^ CD44^high^ T cells). Recipient mice were pretreated with 200 mg/kg bodyweight cyclophosphamide 1 week before the cell transfer. The animals were sacrificed 6 weeks later for end‐point analyses

Six weeks after cell injection, the mice were sacrificed, serum was collected, and the pancreas, liver and kidneys were cryo‐ and paraffin‐embedded for further analyses. The workflow for the adoptive transfer is illustrated in Figure 1.

### 2.4 Isolation, expansion and adoptive transfer of regulatory T cells

For the transfer of *T*_regs_, 27 ± 0.1‐week‐old female MRL/MpJ mice were used as donors of splenocytes (protocol as described above in *Isolation of splenocytes and cell culture conditions*). The isolation of T cells from unpurified splenocytes was performed on the same day using the CD4^+^CD25^+^ Regulatory T Cell Isolation Kit, mouse (130‐091‐041; Miltenyi Biotec) following the manufacturer's instructions.

CD4^+^CD25^+^ T cells were cultured using the *T*_reg_ Expansion Kit, mouse (130‐095‐925; Miltenyi Biotec). Briefly, 2 × 10^6^ cells/mL were dissolved in splenocyte culture medium supplemented with 2000 U/mL interleukin (IL)‐2 (recombinant, murine; Immunotools, Friesoythe, Germany) and sowed into 24‐well plates applying 200 μL/well. Afterwards 200 μL of 6 × 10^6^ CD3/CD28 MACSiBeads/mL in splenocyte culture medium plus 2000 U/mL IL‐2 was added to each well. On day 1, 400 μL/well of splenocyte culture medium, supplemented with 2000 U/mL IL‐2, was added to each well. Half of the medium was replaced with fresh medium and IL‐2 on days 3 and 5. On day 7, the MACSiBeads were removed employing the MACSiMAG Seperator (Miltenyi Biotec) and cells were sowed out again with fresh beads. The expansion was repeated for another week.

After a total of 14 days, the beads were removed again and the cells were resuspended in PBS, pH 7.4. 2.5 × 10^6^ cells in a volume of 200 μL were injected into the tail vain of 30 ± 0.2‐week‐old female MRL/MpJ mice (pretreated with cyclophosphamide as described above; for workflow, see Figure 1). Mice (29 ± 0.2 weeks old) that were not treated with cells served as controls. End‐point analyses for both groups were carried out 6 weeks after cell injection.

### 2.5 Histology and immunohistochemistry

Paraffin‐embedded pancreatic sections were stained with haematoxylin and eosin (H&E), and pathological changes were graded on a semi‐quantitative scale from 0 (none) to 4 (severe) as previously described (H&E‐score).^9^, ^10^, ^12^, ^13^ Briefly, severity of pancreatic lesions was quantified as follows: 0, no pathological changes; 1, minimal infiltration of periductal tissue with mononuclear cells; 2, moderate periductal infiltration with mononuclear cells associated with beginning parenchymal destruction; 3, severe periductal inflammation and/or progressive parenchymal destruction; 4, diffuse mononuclear cell infiltrates, extended destruction of pancreatic tissue and replacement by adipose/fibrotic tissue. H&E staining was also performed on sections of paraffin‐embedded liver or kidney tissue of recipient MRL/MpJ mice. The occurrence of inflammatory foci in these organs was assessed in a qualitative manner.

As a complementary method, immunohistochemistry was performed on 6 μm thick cryosections of pancreatic tissue. Immunohistochemistry was carried out using an avidin‐biotin‐peroxidase (ABC) system (Vector Laboratories, Burlingame, CA, USA). The slides were then counterstained with Mayer's hemalum solution, dehydrated by two short incubations in ethanol and xylene each and embedded in Pertex (MEDITE, Burgdorf, Germany). To define the composition of the inflammatory regions, the following antibodies were used: rat anti‐mouse CD3 (BD Biosciences, Heidelberg, Germany), rat anti‐mouse CD4 (Immunotools), rat anti‐mouse CD8‐β (Santa Cruz Biotechnology, Dallas, TX, USA), rat anti‐mouse CD44 (Thermo Fisher Scientific, Darmstadt, Germany) and biotinylated goat anti‐rat IgG antibody (Vector Laboratories). Extension and density of the specifically stained lymphocytic infiltrates were assessed in a semi‐quantitative manner. Therefore, scores that resembled the H&E‐based scoring system were applied as follows (CD3‐score): 0, no infiltrate; 1, minimal infiltration of periductal tissue; 2, moderate periductal infiltration and beginning infiltration of parenchyma; 3, extensive and multifocal parenchymal and periductal infiltrates; 4, dense and confluent infiltrates throughout the entire section. The so‐termed *AIP‐score* for each animal represents the highest score that was obtained by the evaluation of H&E and CD3 stains, through the assessment of at least 8 pancreatic sections per mouse.

### 2.6 Flow cytometric analysis of T cell subpopulations

Unpurified splenocytes or isolated subpopulations were subjected to flow cytometric analyses to validate the quality of the cells. Prior to staining, the Fc receptors were blocked by preincubation with anti‐CD16/CD32 antibodies (Biolegend, San Diego, CA, USA) for 10 minutes on ice. Surface staining was accomplished by incubating the cells with fluorochrome‐conjugated specific antibodies for 20 minutes in the dark on ice. The following antibodies (all purchased from Miltenyi Biotec) were employed: anti‐CD3‐FITC (130‐102‐496), anti‐CD4‐FITC (130‐102‐541), anti‐CD4‐PE (130‐102‐619), anti‐CD8‐PE (130‐102‐595), anti‐CD19‐FITC (130‐092‐042), anti‐CD25‐APC (130‐102‐787), anti‐CD44‐APC (130‐102‐563), anti‐CD62L‐PE (130‐102‐907).

Intracellular staining of FoxP3 was performed using an anti‐FoxP3‐PE antibody (130‐098‐119; Miltenyi Biotec) and the FoxP3 Staining Buffer Set (130‐093‐142; Miltenyi Biotec) following the given instructions.

Flow cytometric analyses were run on a FACS Verse (BD Biosciences) or FACS Calibur (BD Biosciences). A total of 10 000 events per sample were acquired and data were evaluated using the FACS Suite or CellQuest Pro software (both BD Biosciences).

### 2.7 Statistical evaluations

Data were analysed using the IBM SPSS Statistics 22.0. Values are expressed as mean ± standard error of mean (SEM) for the AIP‐scores of the different mouse cohorts as well as for affected livers and kidneys and the age of the animals. Statistical significance was checked using the Kruskal‐Wallis test followed by the Mann‐Whitney *U*‐test and Bonferroni's post hoc test. *P* < .05 (Bonferroni‐adjusted) was considered to be statistically significant.

## 3 RESULTS

### 3.1 Adult MRL/MpJ mice spontaneously develop an AIP

**Figure 2:**
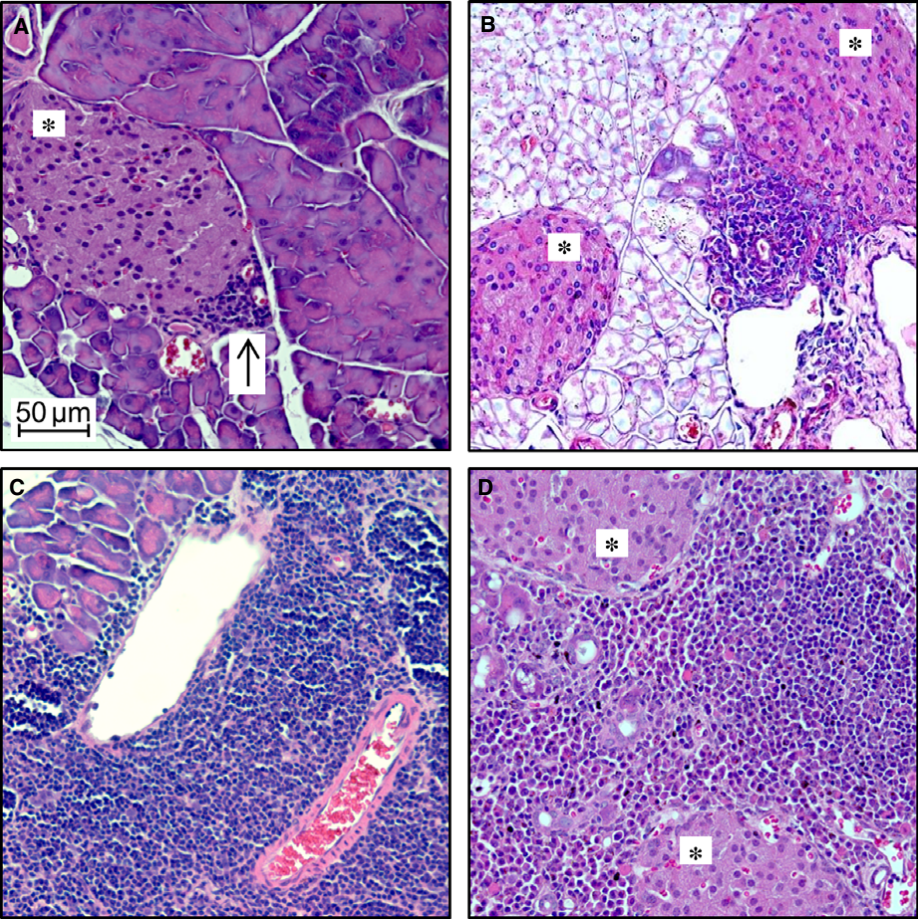
H&E staining of pancreatic tissue from donor MRL/MpJ mice. Shown are exemplary microphotographs of H&E stained pancreatic sections from donor animals. Small areas of infiltrating immune cells (indicated by an arrow) can be found in (A), showing an AIP‐score of 1. The AIP‐score increases with a greater extent of infiltrating cells (B = AIP‐score 2, C = AIP‐score 3, D = AIP‐score 4). The islets of Langerhans (*) stay largely unaffected throughout progression of the disease, while destruction of the parenchyma takes place in animals with a more severe AIP

Female MRL/MpJ mice spontaneously develop AIP at an age of at least 6 months.^12^, ^13^ To perform an adoptive transfer of lymphocytes from adult (sick) to young (still healthy) mice, we had to ensure the breakout of the disease in the donor animal groups. In this study, we used adult female MRL/MpJ mice (as indicated in Table 1) for splenic cell isolation. The severity of the AIP was evaluated by scoring H&E stained pancreatic tissue (Figure 2) and CD3 stained sections (data not shown) in a semi‐quantitative manner employing scores that ranged from 0 to 4.^9^, ^10^, ^13^ The average AIP‐score for all groups of adult donors was approximately 3 (Table 1), representing a severe inflammation with parenchymal destruction. Adult mice without a pancreatic phenotype were disregarded as donors of lymphocytes. For comparison, we also employed one group of young healthy female donors for splenocyte isolation (Table 1).

### 3.2 CD3^+^ T cells effectively transfer murine AIP

**Figure 3:**
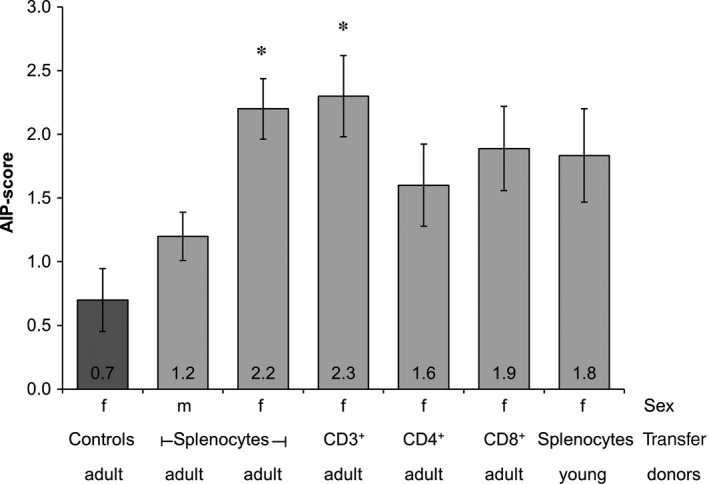
Pancreatic AIP‐scores of MRL/MpJ recipient mice. Splenocytes were obtained from adult (sick) and young (healthy) female MRL/MpJ donor animals as indicated, and subpopulations (CD3^+^, CD4^+^ or CD8^+^ T cells) were isolated 3 days later. Either unpurified splenocytes or the indicated subpopulations were transferred into young recipient mice. Age‐matched MRL/MpJ mice without cell transfer served as controls (darker bar). Six weeks after cell injection, the mice were sacrificed, the pancreata were CD3 and H&E stained and subjected to semi‐quantitative evaluation (scores from 0 to 4). Data are presented as mean ± SEM (n = 6‐10 per group; Table 1), *P < .05 vs untreated controls

For the adoptive transfer of splenocytes, the cells acquired from donor MRL/MpJ mice were cultured for 3 days before CD3^+^, CD4^+^ or CD8^+^ T cells were purified. All isolations lead to highly pure cell populations (Figure S1). Either one of the isolated subpopulations or unpurified splenocytes were then transferred into young and still healthy female MRL/MpJ recipient mice (Table 1). Additionally, unpurified splenocytes were also transferred into male recipient mice. Young female MRL/MpJ mice that were not treated with cells served as a control group. Six weeks after the cell injection, the AIP‐score of the recipient mice was evaluated (Figure 3).

As expected, both animals of the control group and male recipient mice exhibited autoimmune lesions at a low frequency only (average AIP‐scores: control group 0.7 ± 0.2, male recipients 1.2 ± 0.2). In contrast, the transfer of unpurified splenocytes into female MRL/MpJ mice induced the development of inflammatory foci within the pancreatic tissue of recipient mice (average AIP‐score: 2.2 ± 0.2; *P* = .016 vs untreated controls).

Interestingly, CD3^+^ T cells induced an AIP in recipient mice as effective as unpurified splenocytes (average AIP‐score: 2.3 ± 0.3; *P* = .032 vs untreated controls). The transfer of CD4^+^ and CD8^+^ T cells lead to average AIP‐scores of 1.6 ± 0.3 and 1.9 ± 0.3, respectively. When unpurified splenocytes from young mice without a pancreatic phenotype at the time of cell isolation were transferred, an average AIP‐score of 1.8 ± 0.4 was obtained. The three latter scores were not significantly higher than the score of untreated controls.

### 3.3 Composition of inflammatory foci

**Figure 4:**
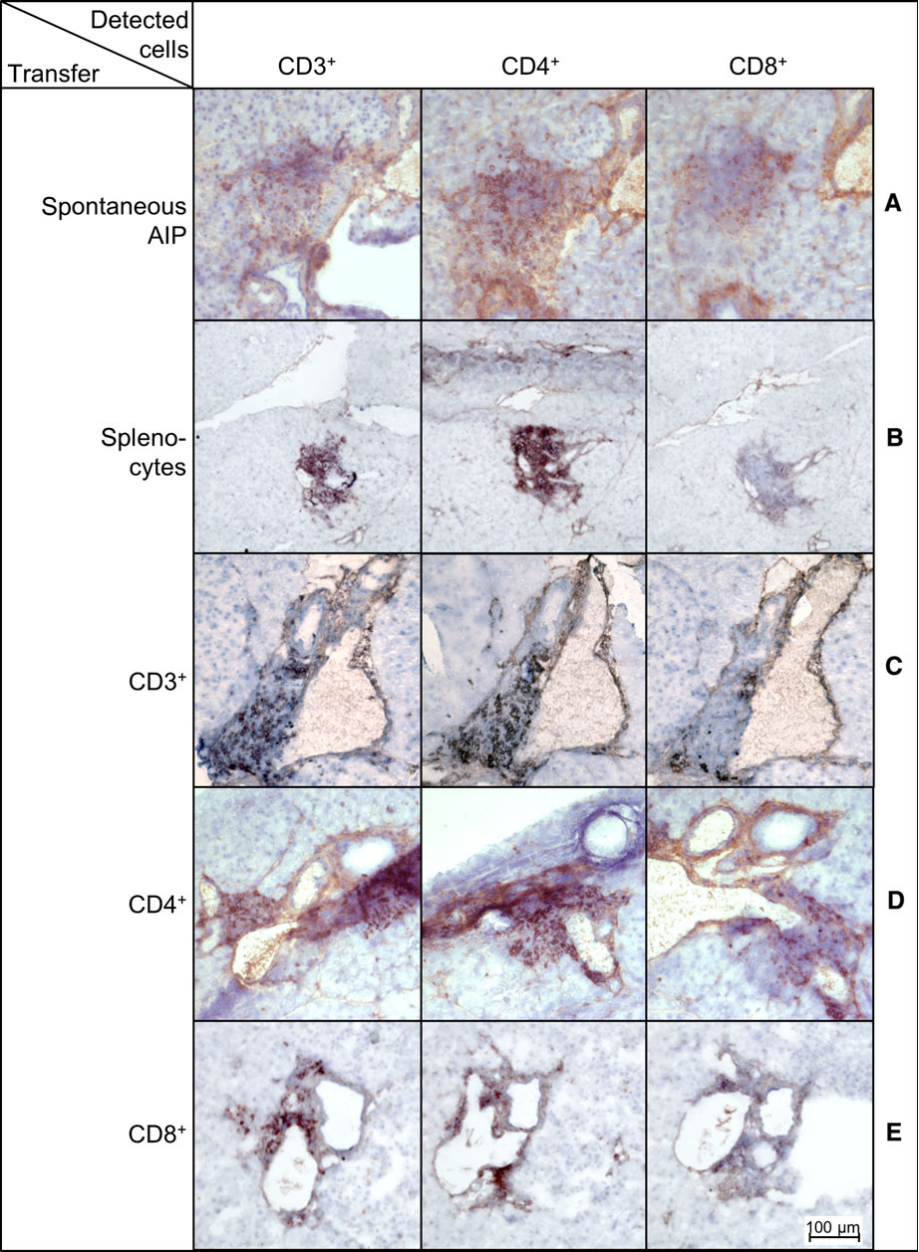
Immunohistochemical staining of pancreatic tissue from MRL/MpJ recipient mice treated with different splenic cell populations. Young MRL/MpJ were injected with different splenic cell populations obtained from adult donor animals. Immunohistochemistry was performed on 6 μm thick pancreatic sections of recipient mice 6 weeks after cell injection. Exemplary pictures are shown for animals with spontaneous AIP (A) or animals treated with unpurified splenocytes (B), CD3^+^ T cells (C), CD4^+^ T_helper_‐cells (D) or CD8^+^ cytotoxic T cells (E). CD3, CD4 and CD8 were stained in serial sections to evaluate the composition of the autoimmune infiltrates

Pancreatic inflammatory foci of MRL/MpJ mice with spontaneous AIP mainly consist of CD3^+^ T cells, with CD4^+^ T cells being predominant over CD8^+^ T cells (Figure 4A).^10^, ^12^, ^13^, ^16^ Autoimmune foci that were induced by the transfer of unpurified splenocytes (Figure 4B), CD3^+^ T cells (Figure 4C), CD4^+^ T cells (Figure 4D) and CD8^+^ T cells (Figure 4E) also largely consisted of CD3^+^ T cells, and higher numbers were observed for CD4^+^ T cells than for the CD8^+^ counterpart.

Thus, our immunohistochemical investigation revealed no differences in the composition of the inflammatory foci of MRL/MpJ recipient mice, independent of the kind of transferred cells.

### 3.4 Other organ involvements

**Figure 5:**
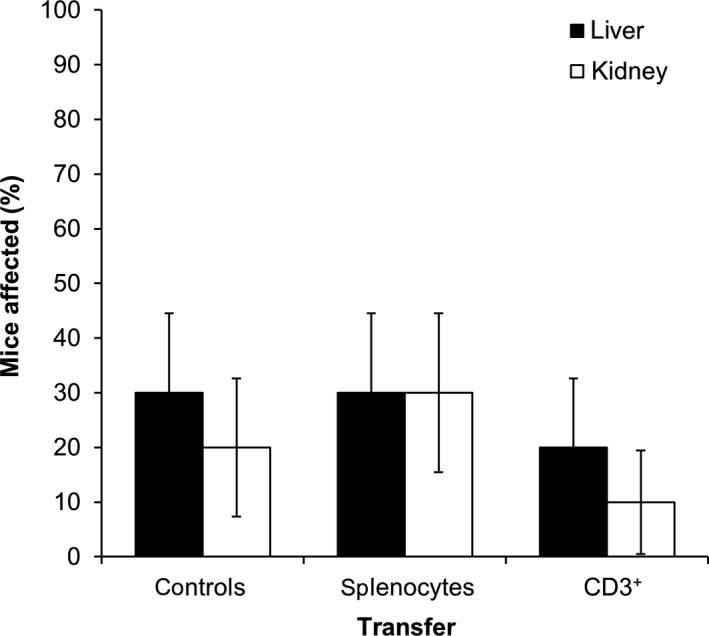
Presence of autoimmune foci in liver and kidney of MRL/MpJ recipient mice. Unpurified splenocytes or CD3^+^ T cells were obtained from adult MRL/MpJ mice and adoptively transferred into young recipient mice. Age‐matched MRL/MpJ mice without cell transfer served as controls. Six weeks after injection, liver and kidney were paraffin‐embedded and subjected to H&E staining. The presence of autoimmune foci within the tissue was examined. Data are presented as mean ± SEM (n = 10 per group). There were no significant differences among the groups

AIP of MRL/MpJ mice is occasionally accompanied by autoimmune lesions in liver and kidney (Ref. 9 and Figure 5). Investigating control mice and recipients of unpurified splenocytes and CD3^+^ T cells from adult mice with AIP, we observed autoimmune foci in these organs in 10%‐30% of the animals (Figure 5 and Figure S2). There were no significant differences between treated mice and controls. Therefore, under the given experimental conditions, the injected cells did not have an impact on other vulnerable organs than the pancreas.

### 3.5 CD4^+^CD44^high^ memory T cells transfer AIP with high efficiency

To gain further mechanistic insights, we employed CD4^+^CD44^high^ memory T cells for additional cell transfer experiments. Splenocytes were isolated from adult female MRL/MpJ mice (average AIP‐score: 3.0 ± 0.2; Table 1) and cultured for 3 days, prior to the isolation of CD4^+^CD44^high^ T cells (for cell purity, please refer to Figure S1). To accommodate for the lower yield, the number of transferred cells was reduced to 2 × 10^6^ cells. Another cohort of mice received the same (reduced) number of unpurified splenocytes, enabling a direct comparison of the findings.

**Figure 6:**
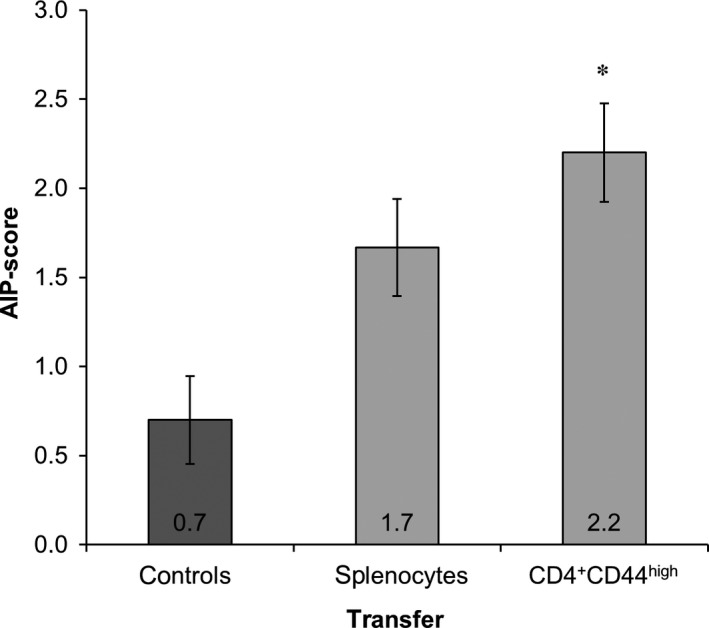
Pancreatic AIP‐score of MRL/MpJ mice treated with CD4^+^ CD44^high^ memory T cells. Splenocytes were obtained from adult female MRL/MpJ donor animals and CD4^+^CD44^high^ T cells were isolated 3 days later. Subsequently, 2 × 10^6^ cells (unpurified splenocytes or CD4^+^CD44^high^ T cells) were transferred into young, female recipient mice. Age‐matched MRL/MpJ mice without cell transfer served as controls (darker bar; same controls as in Figure 3). Data are presented as mean ± SEM (n = 9‐10 per group), *P < .05 vs untreated controls

As shown in Figure 6, unpurified splenocytes induced an AIP with an average AIP‐score of 1.7 ± 0.3, which was only by trend higher than the AIP‐score of 0.7 ± 0.2 for the control group. Notably, the highest average AIP‐score in this experimental series was observed for MRL/MpJ mice receiving CD4^+^CD44^high^ memory T cells (average AIP‐score: 2.2 ± 0.3; *P* = .024 vs untreated controls; Figure 6). Similar to the other immune cells (Figure 4), adoptively transferred CD4^+^CD44^high^ memory T cells did not change composition of the inflammatory infiltrates of MRL/MpJ recipient mice (Figure S3), suggesting a quantitative enhancement, rather than a qualitative modification, of the immune response.

### 3.6 Effects of regulatory T cells on the course of AIP

Next, we asked if transferred *T*_regs_ are capable of inhibiting the progression of spontaneous AIP in mice. Hence, CD4^+^CD25^+^ *T*_regs_ were isolated from the spleens of 27 ± 0.1‐week‐old female MRL/MpJ mice (average AIP‐score: 2.6 ± 0.2), expanded for 2 weeks (for cell purity, please refer to Figure S1) and transferred into female MRL/MpJ mice (2.5 × 10^6^ cells). Recipient mice were at the advanced age of 30 ± 0.2 weeks, when spontaneous AIP occurs at a high frequency.^12^

**Figure 7:**
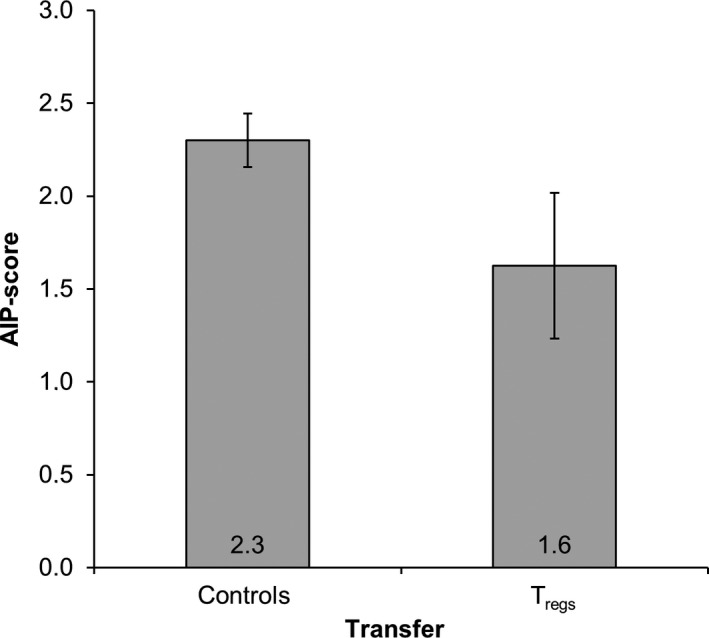
Pancreatic AIP‐score of MRL/MpJ mice treated with regulatory T cells. CD4^+^ CD25^+^ *T*_regs_ were isolated from adult female MRL/MpJ mice (27 ± 0.1 weeks old) and expanded for 2 weeks. Afterwards 2.5 × 10^6^ cells were transferred into female MRL/MpJ mice at an advanced age of 30 ± 0.2 weeks, where spontaneous AIP occurs at a high frequency.^12^ Age‐matched control mice did not receive cells. Six weeks after the injection, pancreatic tissue was CD3 and H&E stained and scored in a semi‐quantitative manner (scores from 0 to 4). Data are presented as mean ± SEM (n = 8‐10 per group). There was no significant difference between the two groups

The AIP‐scores for the injected mice and a control group (no cell transfer) are shown in Figure 7. Untreated control mice presented with an average AIP‐score of 2.3 ± 0.1. The transfer of *T*_regs_ reduced the average AIP‐score to 1.6 ± 0.4. This effect, however, was statistically not significant (*P* = .274 vs controls).

## 4 DISCUSSION

The immunopathogenesis of AIP, a rare form of CP, is largely unknown. In particular, the role of cellular autoimmunity has not been studied in detail. The results of this study identify two lymphocyte subpopulations that efficiently trigger AIP in MRL/MpJ mice, an experimental model of the disease.

Kanno et al^12^ showed the ability of unpurified splenocytes to transfer AIP. Our data confirm and expand this finding by indicating that splenocytes derived from AIP‐prone but still healthy MRL/MpJ mice are less potent than splenocytes from diseased animals, since only in the latter case a significantly increased AIP‐score was observed.

Furthermore, our results revealed that CD3^+^ T cells induce an AIP as efficient as unfractionated immune cells when adoptively transferred from adult and sick MRL/MpJ mice into young and still healthy animals of the same strain. Consequently, cellular immunity mediated by T cell‐dependent processes is essential and sufficient for the transfer of the disease under the given experimental conditions. Evidence for T cell‐mediated processes has also been found in experimental AIP in rats before: Amylase‐specific CD4^+^ T cells could induce AIP in adoptive transfer experiments.^17^ Accordingly, T cells play a key role in cellular immune processes in different experimental models of AIP. In the human situation, the presence of T cells within pancreatic parenchyma has also been described.^8^, ^18^, ^19^

Moreover, CD4^+^CD44^high^ memory T cells were sufficient to transfer murine AIP. Strikingly, the average AIP‐score for recipient mice treated with memory T cells was as high as the AIP‐score for recipient mice treated with CD3^+^ T cells, although the number of transferred cells was less than the half. The studies on the role of CD4^+^CD44^high^ T cells were encouraged by the results of previous investigations of our group: Genotype‐phenotype correlations studies in AIP‐susceptible mice lead to the mapping of quantitative trait loci (QTLs) containing putative susceptibility genes for AIP.^16^ Interestingly, the relative frequency of CD4^+^CD44^+^ splenocytes and the development of AIP were found to be controlled by overlapping QTLs.^15^, ^16^ Additionally, the relative frequency of CD4^+^CD44^high^ memory T cells in the spleen correlated with the severity of AIP.^15^ The unique role of CD4^+^CD44^high^ memory T cells in murine AIP is underscored by the fact that they were the only type of immune cell that fulfilled both criteria (overlapping QTLs with AIP and correlation with disease severity). CD4^+^CD44^high^CD62L^low^ effector memory T cells (*T*_EM_) are, in contrast to naïve T cells, antigen‐primed and thus deliver T cell memory.^20^ *T*_EM_ are located in lymphoid and non‐lymphoid tissue and provide immediate local immune response.^20^, ^21^ With the presented work, we demonstrate a functional role of CD4^+^CD44^high^ memory T cells in the pathogenesis of autoimmune‐related experimental pancreatitis.

The role of CD4^+^ and CD8^+^ T cells in AIP has been discussed before.^3^, ^8^, ^9^, ^16^ Interestingly, neither CD4^+^ nor CD8^+^ T lymphocytes alone were sufficient to induce AIP in healthy (but susceptible) recipients. Together, these findings suggest a contribution of both types of T cells to the exhibition of the disease phenotype.

Regulatory T cells are immune cells with largely inhibitory characteristics.^11^ The specific role of these cells in AIP, however, remains to be elucidated. In MRL/MpJ mice, the therapeutic effect of rapamycin could be linked to the activation of *T*_regs_, which suppress the activity of effector T cells.^9^ This suppressive effect of *T*_regs_ on T cells could also lead to an inhibition of the progression of AIP in vivo. However, under the given experimental conditions, the transfer of activated *T*_regs_ alone was not sufficient to attenuate AIP in MRL/MpJ mice, suggesting that the availability of these cells was not a limiting parameter in our model. Furthermore, the stage of the disease at the time of *T*_reg_ application as well as the specific protocol of cell application needs to be considered as influencing factors.

The MRL/MpJ model of spontaneous AIP that was used in this study mimics the human disease, specifically type 1, in some important regards. Thus, inflammation starts from the exocrine tissue, involves destruction of acinar architecture and increased deposition of extracellular matrix, and leaves the islets largely unaffected. Moreover, dense infiltrates of immune cells, such as T cells and activated B‐cells/plasma cells, are characteristic of both the human and the murine disease.^3^, ^13^, ^16^, ^22^ As a major limitation of any mouse model of AIP, the lack of IgG4 has to be taken into account. The relevance of our findings for the human situation therefore needs to be studied further.

While this study was dedicated to the analysis of the role of different T cell populations, previous experimental studies by us and others have implicated macrophages (particularly, of the M1 subtype) and neutrophils into the pathogenesis of the disease.^9^, ^10^, ^12^, ^13^, ^16^ Specifically, it has been proposed that activated macrophages may induce destruction of pancreatic parenchyma directly or via antibody‐dependent cellular cytotoxicity.^12^ Neutrophils may be activated by endogenous danger signals, such as damage‐associated molecular patterns and autoantibodies, and trigger activation of plasmacytoid dendritic cells through neutrophil‐derived structures termed neutrophil extracellular traps (NETs).^22^ Induction and progression of AIP in mice and men, therefore, depend on a complex interplay between innate and adaptive immune responses that remains to be further elucidated.

In summary, the results of this study provide new insights into the immunopathogenesis of experimental AIP by showing that both CD3^+^ and CD4^+^CD44^high^ T cells effectively transfer AIP from sick MRL/MpJ mice to healthy, but AIP‐susceptible individuals. While our data clearly support a key role of T cells in experimental AIP, they do not address the involvement of autoantibodies to disease induction and progression. As both cellular and humoral immune reactions are likely to contribute to the pathogenesis of human AIP, the interplay between these processes is subject of follow‐up studies of our laboratory.

## CONFLICT OF INTEREST

The authors confirm that there are no conflict of interests.

## AUTHOR CONTRIBUTIONS

LE performed the experiments and wrote the manuscript with the assistance of RJ. SR and RJ participated in the cell culture and animal experiments. All authors contributed to the design of the experiments, evaluated the data and approved the manuscript.
