# Supplementary material for: Adoptive transfer of CD3+ T cells and CD4+ CD44high memory T cells induces autoimmune pancreatitis in MRL/MpJ mice
Source: J Cell Mol Med. 2018 Jan 31;22(4):2404–12. doi: 10.1111/jcmm.13537 (PMC5867153; doi:10.1111/jcmm.13537)
Supplement: Supplementary file 1 [file JCMM-22-2404-s001.doc]

**SUPPORTING INFORMATION**

**Fig. S1. Flow cytometric analyses of transferred T-cell subpopulations.** Different T-cell subpopulations were isolated from adult MRL/MpJ mice. Shown are representative dotplos of all groups.

Unpurified splenocytes, CD3^+^, CD4^+^ and CD8^+^ T-cells were surface stained with
anti-CD3-FITC, anti-CD4-PE, anti-CD8-PE and anti-CD19-FITC in order to validate the purity of the populations. More than 80 % of the unpurified splenocytes were positive for CD3 (R6+R7; **a**), and about 40 % of the splenocytes expressed the B-cell marker CD19
(R10; **a**). The percentage of CD3-positive cells increased to over 90 % when the CD3 T-cell isolation kit was used (R6+R7; **b**). About 50 % of the isolated T-cells were positive for CD4 and CD8 each. Less than 10 % expressed the B-cell marker CD19 (R10; **b**). The isolation of CD4^+^ (**c**) and CD8^+^ (**d**) T-cells lead to populations with a purity of over 90 %
(R6 in **c** = CD4-PE; R6 in **d** = CD8-PE). The percentage of CD19-positive cells was again reduced to less than 10 % (R10; **c** and **d**).

CD4^+^CD44^high^ memory T-cells were stained with anti-CD4-FITC, anti-CD44-APC and anti-CD62L-PE in a triple staining. All cells were negative for CD62L and about 70 % were double positive for CD4 and CD44. The remaining 30 % were still positive for CD44 (**e**).

The population of T_regs_ was surface stained with anti-CD4-FITC and anti-CD25-APC, showing that about 85 % were double positive. Additionally, intracellular FoxP3 was stained. About 25 % of the cells were positive for FoxP3 (**f**).

**Fig. S2. H&E staining of liver and kidney tissue from MRL/MpJ recipient mice.** Shown are exemplary microphotographs of H&E stained liver (**a**) and kidney (**b**) sections from MRL/MpJ mice. Areas of infiltrating immune cells (indicated by an arrow) can be found in both organs, next to healthy tissue.

**Fig. S3. Immunohistochemical staining of pancreatic tissue from MRL/MpJ recipient mice treated with CD4^+^CD44^high^ memory T-cells.** Young MRL/MpJ were injected with CD4^+^CD44^high^ T-cells from adult donor animals. Immunohistochemistry was performed on
6 µm thick pancreatic sections of recipient mice six weeks after cell injection. Exemplary pictures are shown for animals with spontaneous AIP (**a**) and animals treated with unpurified splenocytes (**b**) or CD4^+^CD44^high^ memory T-cells (**c**). CD3, CD4, CD8 and CD44 were stained in serial sections in order to evaluate the composition of the autoimmune infiltrates.


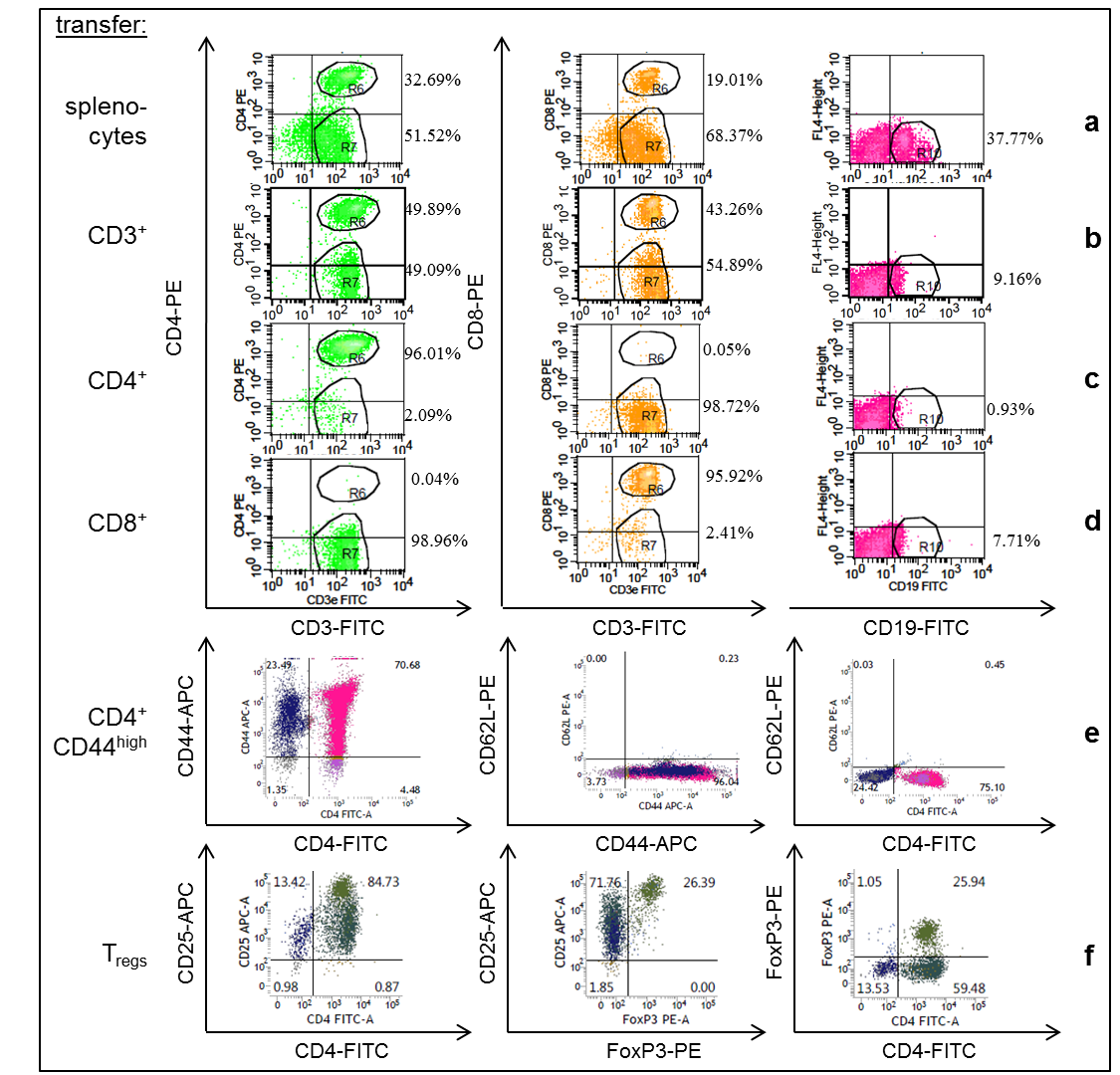
**Fig. S1**

**Fig. S2**

**
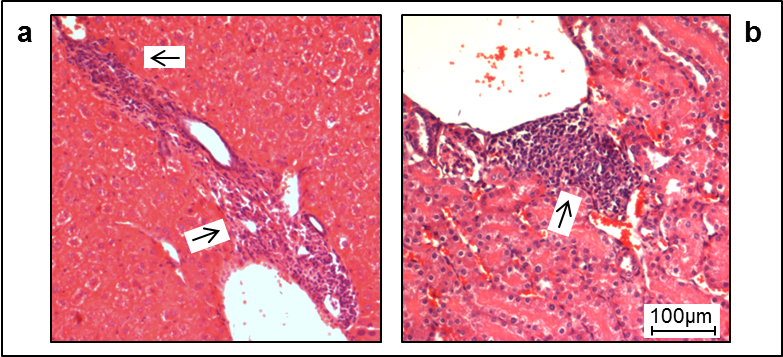
**

**Fig. S3**

**
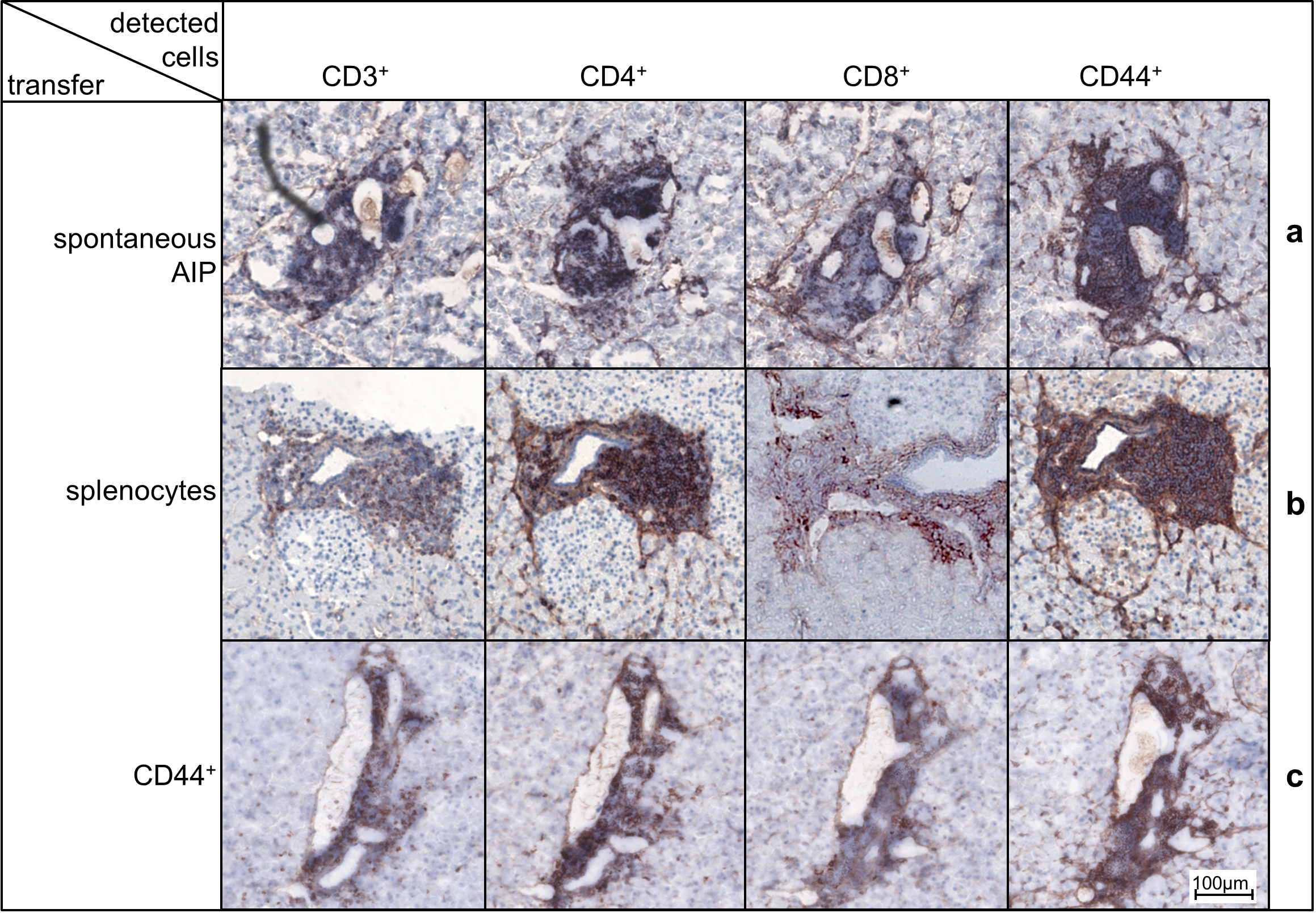
**
